# Genetic diversity analysis of papaya leaf distortion mosaic virus isolates infecting transgenic papaya “Huanong No. 1” in South China

**DOI:** 10.1002/ece3.6800

**Published:** 2020-09-22

**Authors:** Cuiping Mo, Zilin Wu, Hengping Xie, Shuguang Zhang, Huaping Li

**Affiliations:** ^1^ State Key Laboratory of Conservation and Utilization of Subtropical Agro‐bioresources, Guangdong Province Key Laboratory of Microbial Signals and Disease Control College of Agriculture South China Agricultural University Guangzhou China; ^2^ Guangdong Sugarcane Genetic Improvement Engineering Center Institute of Bioengineering Guangdong Academy of Sciences Guangzhou China

**Keywords:** evolutionary mechanism, genetic differentiation, genetically modified papaya, papaya leaf distortion mosaic virus

## Abstract

The commercialized genetically modified papaya “Huanong No. 1” has been utilized to successfully control the destructive virus‐papaya ringspot virus (PRSV) in South China since 2006. However, another new emerging virus, papaya leaf distortion mosaic virus (PLDMV), was found in some PRSV‐resistant transgenic plants in Guangdong and Hainan provinces of South China through a field investigation from 2012 to 2019. The survey results showed that “Huanong No. 1” papaya plants are susceptible to PLDMV, and the disease prevalence in Hainan Province is generally higher than that in Guangdong Province. Twenty representative isolates were selected to inoculate “Huanong No. 1,” and all of the inoculated plants showed obvious disease symptoms similar to those in the field, indicating that PLDMV is a new threat to widely cultivated transgenic papaya in South China. Phylogenetic analysis of 111 PLDMV isolates in Guangdong and Hainan based on the coat protein nucleotide sequences showed that PLDMV isolates can be divided into two groups. The Japan and Taiwan China isolates belong to group I, whereas the Guangdong and Hainan isolates belong to group II and can be further divided into two subgroups. The Guangdong and Hainan isolates are far different from the Japan and Taiwan China isolates and belong to a new lineage. Further analysis showed that the Guangdong and Hainan isolates had a high degree of genetic differentiation, and no recombination was found. These isolates deviated from neutral evolution and experienced population expansion events in the past, which might still be unstable. The results of this study provide a theoretical basis for clarifying the evolutionary mechanism and population genetics of the virus and for preventing and controlling the viral disease.

## Graphical abstract

Papaya leaf distortion mosaic virus (PLDMV) has been reported in Japan and Taiwan, but the molecular evolution and population genetic structure of PLDMV have not been reported. This manuscript presents our recent findings that the isolates collected in this study have greatly diverged, indicating that they belong to a new lineage. Further analysis showed a highly genetic differentiation may contribute to difference of the host range.

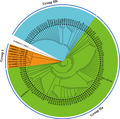

## 1 INTRODUCTION

Papaya leaf distortion mosaic virus (PLDMV) belongs to the species *Papaya leaf distortion mosaic virus* in the genus *Potyvirus* of the family *Potyviridae* and was identified as early as 1954 in the northern area of Okinawa, Japan (Kawano & Yonaha, 1992). It was found in Taiwan China in 1993 (Kiritani & Su, 1999) and spread to the entire island in 2008 (Bau et al., 2008). Xiao et al. (1997) and He et al. (2001), respectively, investigated papaya viruses in papaya planting areas in South China and found only papaya ringspot virus (PRSV) and not PLDMV. Until 2011, PLDMV was reported in some areas of Hainan province, China (Shen et al., 2013; Tuo et al., 2013; Zhao et al., 2018). From 2012 to 2015, we detected the presence of PLDMV in some areas of Hainan and Guangdong provinces and observed that the virus quickly expanded to multiple papaya plantations in South China, causing some fruit farmers to suffer heavy economic losses (unpublished). Papaya plants infected with PLDMV exhibit disease symptoms, such as leaf chlorosis, young leaf distortion and mosaic, water‐soaked oily streaks on the petiole, and ringspots on the fruit (Kawano & Yonaha, 1992). Given that these symptoms, including the subsequent analysis of the transmission vectors and physical and chemical properties, are very similar to PRSV, the virus was initially considered to be PRSV (Maoka et al., 1996). However, further analysis via electron microscopy, serological studies, and whole genome sequencing revealed that the virus is a new species of *Potyvirus* (Bau et al., 2008; Kawano & Yonaha, 1992; Maoka & Hataya, 2005). According to the host range, PLDMV is classified into two types: PLDMV‐P and PLDMV‐C, which are difficult to identify by serological methods but can be distinguished by specific hosts (Maoka & Hataya, 2005). PLDMV‐P mainly infects papaya and few cucurbits (*Cucurbitaceae*), whereas PLDMV‐C does not infect papaya but infects cucurbits (Kiritani & Su, 1999). Similar to PRSV, its genome has a single‐stranded positive‐sense RNA of approximately 10 kb, encoding a polyprotein that is cleaved into 10 mature functional proteins (Yeh et al., 1992). In addition, a short polypeptide (PIPO) is expressed within the P3 cistron by frame shifting (Chung et al., 2008). Among the 11 encoded functional proteins, the coat protein (CP) sequence has been frequently used in strain identification, species classification, and phylogenetic analysis of potyviruses (Chaves‐Bedoya & Ortiz‐Rojas, 2015; Cuevas et al., 2012; Gao et al., 2016; Gibbs & Ohshima, 2010; Martinez, 2014; Wu et al., 2018).

In the 1990s, in order to prevent and control PRSV, American scientists developed antiviral transgenic papaya containing the PRSV CP sequence, which was commercialized in Hawaii in 1998 (Gonsalves, 1998). Later, we obtained the transgenic papaya “Huanong No. 1” containing the PRSV replicase sequence and was approved by the Ministry of Agriculture of China in 2006 for commercial cultivation in South China (Guo et al., 2009; Li et al., 2007; Rao et al., 2012). Since then, transgenic papaya “Huanong No. 1” plants have massively been commercially planted in Guangdong and Hainan provinces and sustained high resistance to PRSV. However, some “Huanong No. 1” plants in several plantations are found to be susceptible in recent years, and most of them are identified to be infected with PLDMV. Current research on PLDMV is only limited to detection and identification, and the molecular evolution and population genetic structure of PLDMV have not been reported. Studying molecular evolution helps to understand the molecular basis of the adaptation, geographic expansion, and emergence of the virus, which are the key to its management and control (Lauring & Andino, 2010).

With the rapid development of sequencing technology, the sequencing analysis of whole genome sequence provides a good condition for the study of molecular evolution mechanism. To understand the genetic diversity and population genetic structure of PLDMV, we investigated and sequenced PLDMV isolates collected from Guangdong and Hainan provinces of South China from 2012 to 2019. We further analyzed the molecular genetic structure of PLDMV in terms of genetic diversity, recombination, phylogeny, selection pressure, and neutral evolution detection. A systematic molecular genetic structure analysis was performed to understand the genetic diversity and population genetic structure of PLDMV in Guangdong and Hainan provinces, clarify the evolutionary mechanism of the virus and the genetic mechanism of the population and further provide theoretical basis for the prevention and control of the viral disease.

## 2 MATERIALS AND METHODS

### 2.1 Field investigation and sample collection

**FIGURE 1:**
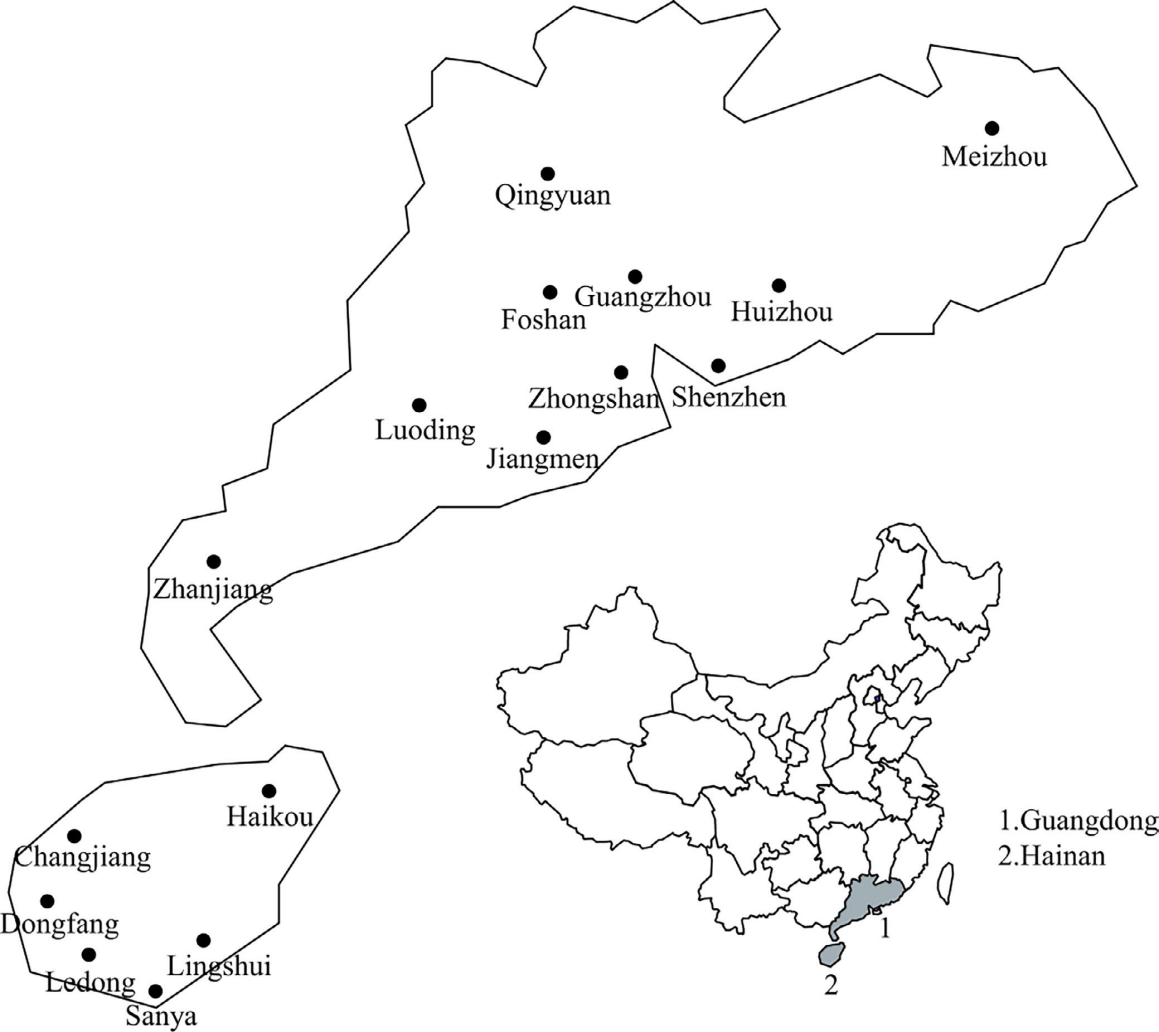
Skeleton drawing map showing the localities of the field investigation and samples collection of papaya leaf distortion mosaic virus isolates in this study

A total of 48 plantations of transgenic “Huanong No.1” papaya from ten counties (Guangzhou, Jiangmen, Zhanjiang, Zhongshan, Foshan, Shenzhen, Qingyuan, Huizhou, Luoding, and Meizhou) in Guangdong Province and six counties (Sanya, Ledong, Dongfang, Changjiang, Haikou, and Lingshui) in Hainan Province were investigated from 2012 to 2019 (Figure 1). Random sampling method was adopted in each plantation, and the disease prevalence was recorded and calculated. A total of 200 infected fresh leaves were sampled, stored on ice‐cold package, and taken back to the laboratory. RT‐PCR and sequencing analysis were employed to identify the PLDMV isolates (Table S1).

### 2.2 Artificial inoculation

According to two types of leaf symptoms (mosaic and distortion), all diseased papaya samples collected in Guangdong Province were divided into two groups. Similarly, the diseased papaya samples collected in Hainan Province were also divided into two groups. 0.1 M phosphate buffer (pH 7.0) was used to grind leaf samples as inoculum. Five representative samples were selected from each group and inoculated the seedlings with 2 leaves of squash cultivar “Yucuixihulu” (*Cucurbita pepo*) and with 3–5 leaves of transgenic papaya cultivar “Huanong No. 1” (Cai & Fan, 1994). Phosphate buffer was administered in the control group. Symptoms were recorded at 7–15 days postinoculation (dpi) from 15 plants per treatment, and the experiment was repeated thrice. The plants were maintained in a greenhouse at 25–28°C under natural light. Total RNAs were extracted from inoculated squash and papaya leaves, and the coat protein sequences of PLDMV isolates were amplified by RT‐PCR and sequenced to compare with the sequences of the original inoculated PLDMV isolates. Results of the experiment were used for verifying Koch's postulates and comparing the isolates' biological characteristics.

### 2.3 Viral RNA extraction, cDNA cloning, and sequencing

Total RNAs were extracted from the collected papaya leaves using the Tiangen Trizol Kit in accordance with the manufacturer's instructions (TianGen). Primers (CP‐F: TCCGCTCTTGATGCTGGCAAA, CP‐R: ATAATATCGAGCGCCGGTGA) were designed on basis of the conserved region sequences of four PLDMV isolates (EU240888.1, EU240889.1, EF675245.1, and AB088221.1) registered in GenBank by using Primer Premier v6.0 software (PREMIER Biosoft International). Full‐length cDNAs were synthesized using PrimeScript™ 1st Strand cDNA Synthesis Kit (Takara). PCR amplification was conducted in a total volume of 50 µl containing 2 µl of template cDNA, 10 µl of 5× PrimeSTAR Buffer (Mg^2+^ Plus), 4 µl of dNTP Mixture (2.5 mM each), 1.5 µl of each primer (10 µM), 0.5 µl of PrimeSTAR HS DNA Polymerase (2.5 U/µl) (Takara), and 30.5 µl of ddH_2_O. The PCR program conditions were as follows: after initial denaturation step at 94°C for 3 min, 35 cycles were performed consisting of three steps: denaturation at 94°C for 30 s, annealing at 55°C for 30 s, and extension at 72°C for 1 min. The final elongation step was performed at 72°C for 5 min. PCR products were separated by 1% agarose gel electrophoresis, extracted, and then purified from the gel with a Tiangen Gel Extraction kit (TianGen). The purified products were cloned into the pMD20‐T vector (Takara) in accordance with the manufacturer's protocol and then transformed into *Escherichia coli* DH5α competent cells. Three positive clones from each transformation were selected and sequenced in both directions by Sangon Biotech (Sangon). All sequences generated in the study were deposited in the GenBank database (Table S1). In addition, 11 PLDMV nucleotide sequences of different isolates from different countries and regions were downloaded from GenBank as the reference sequences (Table S1).

### 2.4 Genetic diversity and population genetic differentiation

The nucleotide sequences of 111 PLDMV isolates were aligned by MAFFT v7.149b software (Katoh & Standley, 2013). Nucleotide sequence identity matrices were calculated using BioEdit software after all gaps were removed (Hall, 1999). The Boxplots of 111 Guangdong and Hainan isolates and three representative isolates (J56P, J199C, and WF) previously reported from Japan and Taiwan China were mapped using R 2.9.1 (R Project for Statistical Computing website). The amino acid sequences of 10 representative isolates from Guangdong and Hainan and three isolates (J56P, J199C, and WF) from Japan and Taiwan China were selected for further analysis of mutation sites. After sequence comparison with NCBI, the conserved regions and mutations were analyzed and edited in Word software.

Haplotype and nucleotide diversities were estimated using DnaSP 5.0 (Librado & Rozas, 2009). Haplotype diversity refers to the frequency and number of haplotypes in the population. Nucleotide diversity estimates the average pairwise differences among the sequences. Genetic differentiation among populations was calculated by *F*_ST_ using Arlequin 3.5 (Excoffier & Lischer, 2010). Genetic differentiation among populations was also calculated by *K*_ST_ and *S*_nn_ using DnaSP 5.0 (Hudson et al., 1992; Librado & Rozas, 2009). The hypothesis of deviation from the null population differentiation was tested by 1,000 permutations of the raw data. The level of gene flow between populations was measured using the *N*_m_ value calculated by DnaSP 5.0 (Librado & Rozas, 2009).

### 2.5 Recombination analysis

The nucleotide sequences of 111 PLDMV isolates from Guangdong and Hainan were subjected to recombination analysis using seven methods (RDP, GENECONV, BOOTSCAN, MaxChi, CHIMAERA, SiSCAN, and 3SEQ) implemented in the RDP v4.71 software package (Martin et al., 2015). The probability of a putative recombination event was corrected by a Bonferroni procedure with a cutoff of *p*‐value <.01. To avoid misidentification, only events supported by at least four of the seven methods were considered to be recombinants.

### 2.6 Phylogenetic analysis

The CP nucleotide sequences of the PLDMV isolates were aligned using the Muscle algorithm (Edgar, 2004) implemented in MEGA 6 (Kumar et al., 2018). A phylogenetic tree of the PLDMV isolates excluding the recombinants was reconstructed by the neighbor‐joining method (Saitou & Nei, 1987) implemented in MEGA 6. At the same time, the sequences of two isolates (DF_HN and LM_HN) previously reported in Hainan Province were retrieved from the GenBank database (JX974555.1 and KT633944.1). Thus, a total of 113 full‐length CP sequences of PLDMV isolates from South China were used for population genetic analysis of the virus. In addition, nine CP sequences from five Taiwan China isolates and four Japan isolates were downloaded from the GenBank database as references (Table S1). The CP sequence of one PRSV isolate (Accession No. KT895257.1) was used as an outgroup. Bootstrap analysis was repeated 1,000 times to evaluate the significance of the internal branches. Intra‐ and intergroup genetic distances were calculated using MEGA 6.

### 2.7 Selection pressure analysis

Selection pressure was estimated by the dN/dS ratio, where dN represents the average number of nonsynonymous substitutions per nonsynonymous site and dS represents the average number of synonymous substitutions per synonymous site. HyPhy 2.10b (Pond & Frost, 2005) was used to identify the nucleotide sites in CP cistrons that may be involved in viral adaptation. Three codon‐based approaches, namely fixed effects likelihood (FEL), internal branches FEL (IFEL), and mixed effects model of evolution (MEME), are included in the HyPhy package (Kosakovsky Pond et al., 2011; Murrell et al., 2012; Pond et al., 2006).

### 2.8 Neutrality test

Tajima's *D*, Fu and Li's *D*, and Fu and Li's *F* values of Arlequin 3.5 (Excoffier & Lischer, 2010) were used to conduct neutrality test on the PLDMV isolates from Guangdong and Hainan to clarify the historical dynamics of both populations. These values assume that all mutations are selectively neutral. Tajima's *D* test identifies evolutionary events, such as population expansion, bottleneck effects, and natural selection, by comparing the numbers of segregating sites and the average number of nucleotide differences (Tajima, 1989). Fu and Li's *D* and Fu and Li's *F* values are sensitive to population expansion and are usually negative when the population is expanded (Fu & Li, 1993).

## 3 RESULTS

### 3.1 Field investigation of the virus infection

In recent years, the disease prevalence in Guangdong province was generally 5%–30% but ≥60% in few seriously diseased areas, whereas that in Hainan province was usually 80% but 90%–100% in some areas. This finding suggests that “Huanong No.1” papaya plants in South China are susceptible to viruses and that the disease prevalence in Hainan province is generally higher than that in Guangdong province. A total of 200 diseased samples showing symptoms of leaf mosaic and malformation, streaks on stems, and petioles and ringspots on fruits were collected from “Huanong No.1” papaya plantations in Guangdong and Hainan provinces during 2012–2019 (Figure 1). All of these samples were identified by RT‐PCR and then by sequence analyses. Results showed that 111 of those samples were infected with PLDMV, including 33 samples from Guangdong Province and 78 samples from Hainan Province (Table 1).

**TABLE 1:** Sample sizes and genetic variation of papaya leaf distortion mosaic virus populations from Guangdong and Hainan of South China based on the CP nucleotide sequences

Region	Sample size	Haplotypes	Haplotype diversity	Nucleotide diversity
GD	33	16	0.816	0.002
HN	78	31	0.908	0.004
All	111	47	0.939	0.005

Note

Haplotype diversity and nucleotide diversity were estimated using DnaSP 5.0.

Abbreviations: All, Guangdong and Hainan isolates; GD, Guangdong isolates; HN, Hainan isolates.

### 3.2 Symptoms of squash and papaya plants inoculated with representative isolates

**FIGURE 2:**
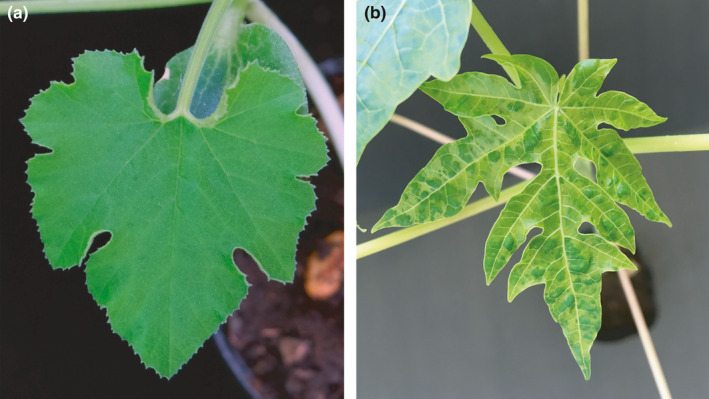
Different types of symptoms developed on *Cucurbita pepo* variety “Yucuixihulu” (a) and transgenic “Huanong No. 1” papaya (b) at 15 days after mechanical inoculation with one representative isolate of papaya leaf distortion mosaic virus, respectively

The inoculation results of three repetition showed that all of the representative isolates from four groups of the samples caused similar viral symptoms in the inoculated plants of papaya. Specifically, in the early stage (10–15 dpi), mosaic, irregular chlorosis, and green islands on the young leaves of papaya were observed. In the later stage (30 dpi), the leaves were gradually deformed in the shape of chicken feet. However, no symptom was observed on the squash plants (Figure 2). The total RNAs were extracted from the inoculated leaves of plants, and the PLDMV CP sequences were amplified by RT‐PCR. The results showed that PLDMV was detected in inoculated papaya but not in squash. The products of RT‐PCR from the inoculated papaya plants were further cloned and sequenced, and the results showed that the nucleotide sequences were essentially coincident with those of the original inoculated isolates. These results indicate that the PLDMV isolates from Guangdong and Hainan provinces can infect transgenic papaya but not squash.

### 3.3 Analysis of sequence variation in the CP regions of PLDMV

**FIGURE 3:**
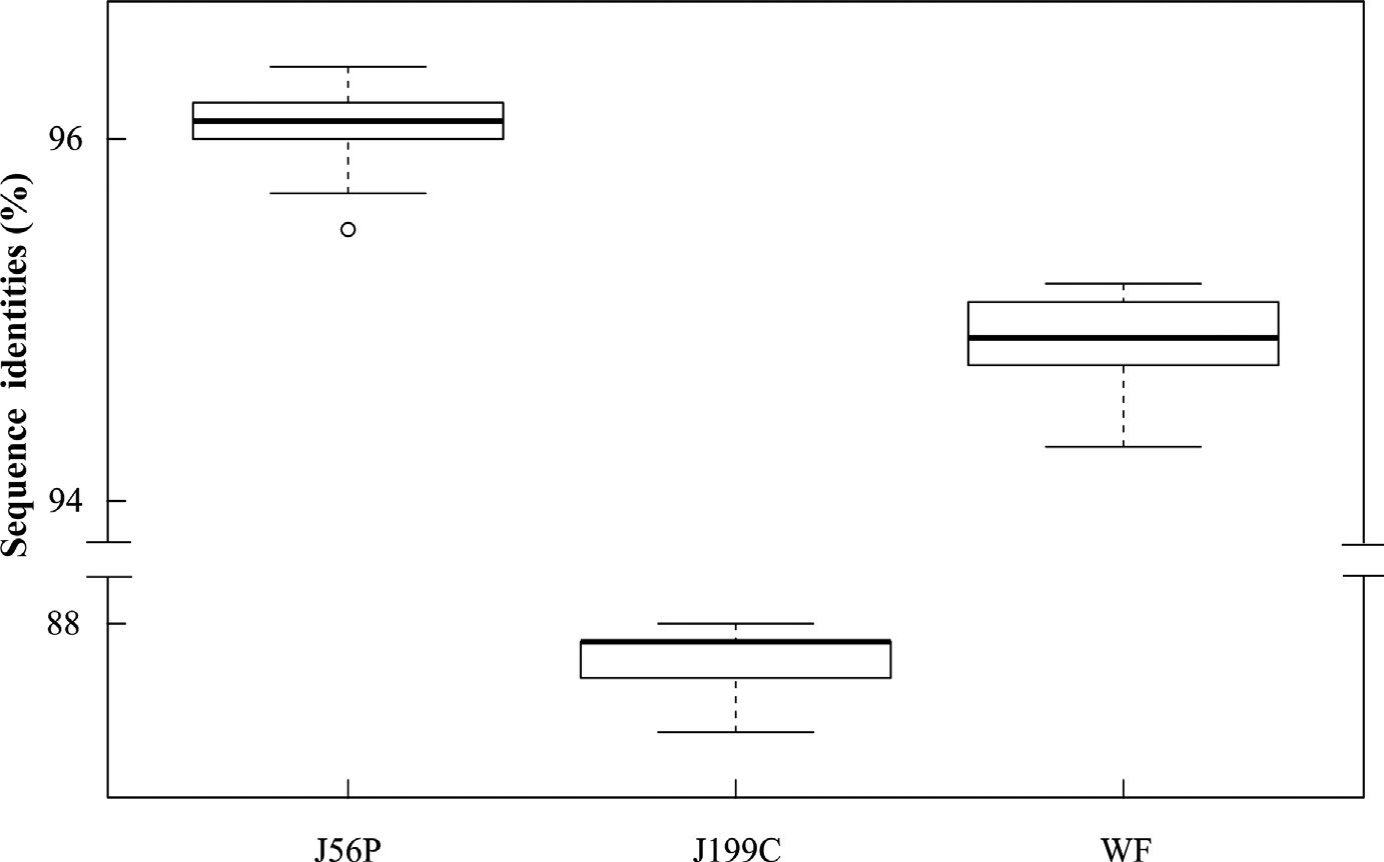
Boxplot graphs showing nucleotide sequence identity scores of coat protein regions of papaya leaf distortion mosaic virus between three representative isolates (J56P, J199C, and WF, previously reported in Japan and Taiwan China) and 111 Guangdong and Hainan isolates. The graphs were mapped using R 2.9.1 (R Project for Statistical Computing website)

**FIGURE 4:**
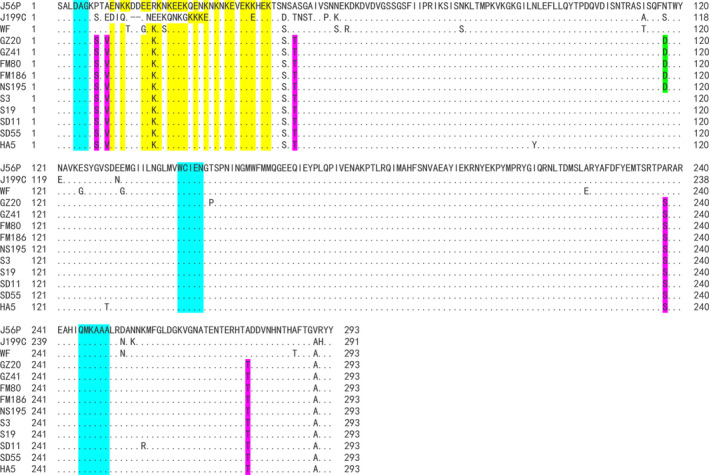
The amino acid sequences of the coat protein of papaya leaf distortion mosaic virus isolates from Japan and Southern China showing the deletions (−) in the J199C isolate, conserved DAG, WCIEN, and QMKAAA domains (blue regions) and mutation sites. J56P and J199C were the previously reported main isolates in Japan, and WF was the main isolate in Taiwan China. GZ20, GZ41, FM80, FM186, and NS195 are Guangdong isolates, while S3, S19, SD11, SD55, and HA5 are Hainan isolates. Different letters in the same column indicate different amino acids in that position. The yellow regions mean the glutamic acid and lysine (EK) repeat patterns, the purple regions mean the different amino acids between this study's isolates and three previously predominant isolates in Japan and South China, and the green region means the different amino acid between Guangdong and Hainan isolates. Multiple alignments were performed with CLUSTALW program included in MEGA X

A total of 111 isolates of PLDMV from Guangdong and Hainan were sequenced, and CP sequences with a length of 879 bp of each isolate were obtained, including 33 in Guangdong and 78 in Hainan. Sequence alignment results showed that the nucleotide sequence identities of the 111 PLDMV isolates from Guangdong and Hainan with three isolates from Japan (J56P and J199C) and Taiwan China (WF) were 95.6%–96.5%, 88.1%–88.8%, and 94.3%–95.2%, respectively. The identities of 33 PLDMV isolates from Guangdong were 84.7%–100%, whereas those of 78 isolates from Hainan were 92.3%–100%. Boxplot analysis showed that the identities between the three isolates (J56P, J199C, and WF) and all other isolates were 86.6%–89.2%, 89.2%–92.4%, and 89.2%–92.1%, respectively (Figure 3). Five representative isolates from Guangdong (GZ20, GZ41, FM80, FM186, and NS195) and five isolates from Hainan (S3, S19, SD11, SD55, and HA5) were selected for comparison of the amino acid sequence of CP with three Japan and Taiwan China isolates (J56P, J199C, and WF). As a result, multiple sites of variation were found (Figure 4). Among them, only J199C lacks two amino acids in “EK” (glutamic acid and lysine) repeat region. The E in the partial region is converted to N, presenting an “NK” (asparagine and lysine) repeat region that is different from other isolates of potyvirus. In addition, the amino acids of 10 isolates at the three sites at the N‐terminus were different from those of J56P, J199C, or WF, that is, from proline (Pro8) to serine (Ser), alanine (Ala10) to valine (Val), and alanine (Ala46) to threonine (Thr). The two sites were changed at the C‐terminal, that is, alanine (Ala237) to serine (Ser) and alanine (Ala277) to threonine (Thr). However, only one site was different (Asn117 and Asp117) between the Guangdong and Hainan isolates. Meanwhile, three conserved DAG, WCIEN, and QMKAAA regions were found in the 13 isolates.

Genetic variation analysis of 111 CP nucleotide sequences from Guangdong and Hainan isolates showed that 47 haplotypes were identified, including 16 from 33 Guangdong isolates and 31 from 78 Hainan isolates (Table 1). The haplotype diversity was 0.939 in all isolates, 0.816 in the Guangdong isolates, and 0.908 in the Hainan isolates, indicating that the genetic diversity of the Hainan population is higher than that of the Guangdong population. The overall nucleotide diversity was 0.005, whereas that of the Guangdong and Hainan isolates was 0.002 and 0.004, respectively. This result further indicates that the genetic diversity of the Hainan population is higher than that of the Guangdong population. In addition, the haplotype diversity of all isolates from Hainan and Guangdong was >0.5, and the nucleotide diversity was <0.005, indicating that the PLDMV population in both regions has high genetic diversity.

### 3.4 Analysis of genetic differentiation

The genetic differentiation of 111 CP sequences of the Guangdong and Hainan isolates showed that *K*_ST_, *S*_nn_, and *F*_ST_ were 0.295, 1.000, and 0.530, respectively (Table 2). The *p* values of these indicators from the outcome of 1,000 Bernoulli trials were 0.000, which show statistically significant differences at the 0.001 level. Hence, the null hypothesis that the two populations from Guangdong and Hainan are not genetically differentiated was rejected. The above three indicator values revealed a significant differentiation between the two PLDMV populations from Guangdong and Hainan. Gene flow detection was performed on the 111 CP sequences of all isolates from Guangdong and Hainan provinces. The results showed that *N*_m_ was 0.220 (Table 2) between two populations from Guangdong and Hainan. This finding indicates that the genetic drift is easy to occur and that gene flow is not frequent between the two populations, thus leading to remarkable genetic differentiation between populations.

**TABLE 2:** Statistical tests for differentiation between two papaya leaf distortion mosaic virus populations from Guangdong and Hainan based on the CP nucleotide sequences

*K*_ST_	*p*	*S*_nn_	*p*	*F*_ST_	*N*_m_
0.295	.000*	1.000	.000*	0.530	0.220

Note

*K*_ST_, *S*_nn_, and *N*_m_ were implemented in DnaSP 5.10, while *F*_ST_ was in Arlequin 3.5. The hypothesis of deviation from null population differentiation was tested by 1,000 permutations of the raw data.

* 0.01 < *p *< .05; **.001 < *p*<.01; ***p < .001.

### 3.5 Recombinant analysis

The RDP software was used to detect the possible recombination events of 111 isolates in Guangdong and Hainan. The results showed that none of the recombination event was supported by at least four of the seven methods. This result indicates that recombination does not occur in all of the Guangdong and Hainan isolates.

### 3.6 Phylogenetic analysis

**FIGURE 5:**
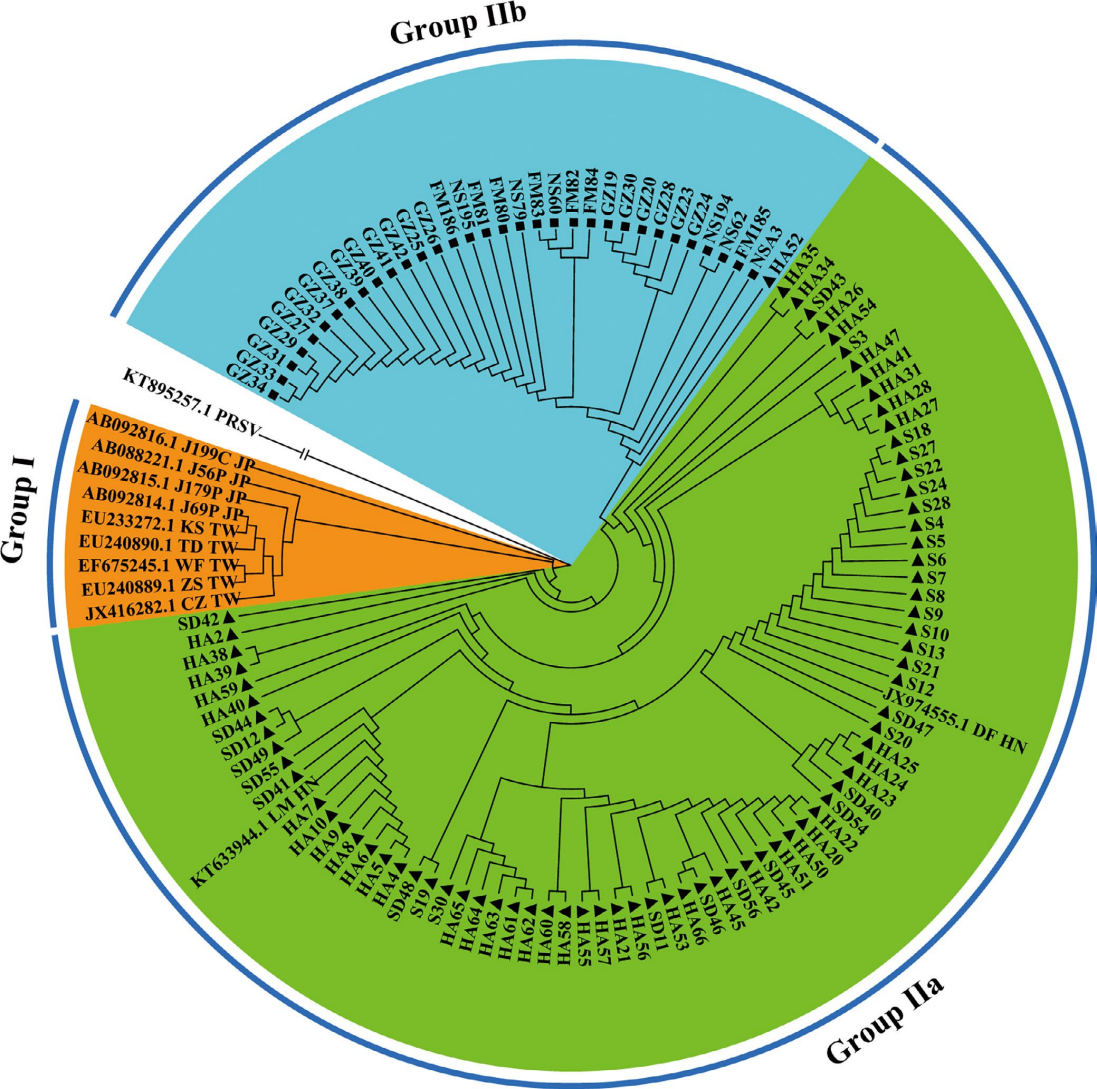
Phylogenetic tree of 111 isolates of papaya leaf distortion mosaic virus from Guangdong and Hainan and 11 isolates from Japan, Taiwan China, and Hainan derived from GenBank was reconstructed by neighbor‐joining method implemented in MEGA6 based on the CP nucleotide sequences. The sequenced PLDMV as reference sequences presented with GenBank Accession number followed the region code: HN for Hainan, TW for Taiwan China and JP for Japan. ▲ is for Hainan, and ■ is for Guangdong

A total of 122 CP nucleotide sequences were used to construct a phylogenetic tree by combining 113 sequences from Guangdong and Hainan isolates with 9 sequences from Japan and Taiwan China isolates downloaded from NCBI (Figure 5). The phylogenetic tree diagram showed that 122 isolates were divided into two groups (Figure 5). Japan and Taiwan China isolates were clustered into group I, whereas Guangdong and Hainan isolates were clustered into group II, which was further divided into two subgroups. Most Hainan isolates were grouped into subgroup I, and all Guangdong isolates were grouped into subgroup II. The genetic distance value was 0.046 ± 0.006 between groups I and II, 0.040 ± 0.005 within group I, and 0.005 ± 0.001 within group II (Table 3). These results implied that the genetic diversity between Guangdong and Hainan isolates (group II) and Japanese isolates and Taiwan China isolates (group I) was greater than each within the groups, suggesting that the genetic diversity between groups is greater than that within the groups. Thus, Guangdong and Hainan isolates (group II) may have differentiated into a new emergent lineage of PLDMV.

**TABLE 3:** Genetic distances between and within variant groups of papaya leaf distortion mosaic virus isolates based on the CP sequences

Phylogroup	Between groups	Within groups
Group I		0.040 ± 0.005
Group II	0.046 ± 0.006	0.005 ± 0.001

Note

The intra‐ and intergroup genetic distances were calculated by MEGA X.

### 3.7 Selection pressures acting on the CP regions of PLDMV isolates

Numerous selection pressure sites (Table 4) were detected in the 111 CP sequences of PLDMV isolates from the Guangdong and Hainan with the FEL, IFEL, and MEME methods in HyPhy package. Zero positive selection and 26 purifying (negative) selection sites were detected by FEL, and one positive and four purifying selection pressure sites were detected by IFEL. The number of purifying selection pressure sites was much higher than that of positive selection pressure sites, indicating that the CP regions of PLDMV isolates are mainly acted under the pressure of purifying selection.

**TABLE 4:** The number of selection pressure sites detected in the 111 CP nucleotide sequences of papaya leaf‐distortion mosaic virus isolates from Guangdong and Hainan

Sites^*^	Purifying		Positive		
	FEL	IFEL	FEL	IFEL	MEME
879	26	4	0	1	1

Note

Sites*, Total number of sites (excluding sites with gaps/missing data). FEL, IFEL, and MEME were estimated by HyPhy package.

### 3.8 Neutrality tests

Although Tajima's D, Fuand Li's D and Fu, and Li's *F* values for two groups from Guangdong and Hainan were all negative, the data were significant or extremely significant (Table 5). The results indicate that PLDMV populations in Guangdong and Hainan deviate from neutral mutation and that the two populations may not be stable.

**TABLE 5:** Neutrality tests of 111 CP nucleotide sequences of papaya leaf distortion mosaic virus isolates from Guangdong and Hainan

Region	Tajima's *D*	Fu and Li's *D*	Fu and Li's *F*
GD	−2.111*	−2.962*	−3.164*
HN	−2.291*	−4.863*	−4.612*
All	−2.238*	−5.605*	−5.023*

Note

Tajima's *D*, Fu and Li's *D*, and Fu and Li's *F* were implemented in Arlequin 3.5.

* 0.01 < *p* <.05; **0.001 < *p*< .01; ***p < .001.

## 4 DISCUSSION

Papaya is an important fruit crop that is widely cultivated in tropical and subtropical regions of countries worldwide (Manshardt, 1992). PRSV is the most widespread and damaging virus that infects papaya and the main factor limiting the development of papaya industry (Mishra et al., 2016; Yeh et al., 2010). The large‐scale commercial cultivation of antiviral transgenic papaya plants has been used to successful control the disease in Hawaii and South China for a long time (Gonsalves, 1998; Li et al., 2007; Mishra et al., 2016; Tecson Mendoza et al., 2008; Yeh et al., 2010). However, recent studies have found that PRSV infects “Huanong No.1” transgenic papaya plants in some regions of South China, and the occurrence tendency increases gradually with time (Zhao et al., 2015). To explain the loss of transgenic papaya resistance against PRSV in South China, Wu et al. (2018) investigated the virus occurrence in “Huanong No.1” papaya plantations in Guangdong and Hainan in 2012–2016. They identified a genetic variation of PRSV and revealed that the formation of new emergent lineage of PRSV may be one of the reasons for the loss of resistance to genetically modified papaya in South China. In this study, we continued to analyze samples of diseased transgenic papaya plants collected in these two areas from 2012 to 2019 and found that another virus, PLDMV, may also be an important factor leading to the loss of resistance to genetically modified papaya in South China.

Maoka and Hataya (2005) classified PLDMV into PLDMV‐P and PLDMV‐C biotypes on the basis of the host range of PLDMV in Japan. PLDMV‐P type can infect *Cucurbitaceae* and papaya, whereas PLDMV‐C type can infect *Cucurbitaceae* and *Nicotiana benthamiana* but not papaya. However, Bau et al. (2008) observed that all PLDMV isolates from Taiwan China can only infect papaya and not other family plants. These results indicate that the Taiwan China isolates are different from PLDMV‐P and C in Japan and may be a new type of PLDMV. In the present study, four groups of PLDMV representative isolates from Hainan and Guangdong provinces of South China were inoculated on squash and transgenic papaya, respectively. No viral symptom was observed, and PLDMV was not detected on squash leaves; however, transgenic papaya plants showed obvious infection symptoms, and PLDMV was detected (Figure 2). These results confirmed, on the one hand, that the isolates we obtained from the samples collecting from Guangdong and Hainan provinces are the agents of the disease and, on the other hand, that the infection characteristics revealed that the isolates from Hainan and Guangdong provinces are similar to the Taiwan China isolates rather than Japan isolates.

As shown in the sequence alignment, the CP nucleotide sequence identity of 111 Guangdong and Hainan isolates in with those of Japan isolates (J56P and J199C) and Taiwan China isolate (WF) were 95.6%–96.5%, 88.1%–88.8% and 94.3%–95.2%, respectively. This result suggests that the 111 isolates are quite different from J199C but are relatively close to J56P and WF. Ten representative isolates from Guangdong and Hainan were further selected for amino acid sequence alignment with J56P, J199C, and WF (Figure 4). We found that J199C lacked two amino acids in the “EK” (glutamic acid and lysine) repeat region, and E was converted to N, showing an “NK” repeat region different from that of other potyviruses. The “EK” region is located on the outer surface of CP (Shukla & Ward, 1989), which is closely related to aphid transmission element (Atreya et al., 1990). Thus, the change of amino acids in the “EK” repeat region may affect the spread of PLDMV by aphids. Given that PRSV has been restricted for a long time by the widely grown commercial anti‐PRSV transgenic papaya in South China, PLDMV may have evolved a new “EK” repeat region suitable for aphid transmission and gradually disseminated and substituted for the original PRSV, which may also be the reason why PLDMV can spread rapidly once it is found in the field. Outside of the “EK” domain, five amino acid sites (three at the N‐terminus and two at the C‐terminal) in the 10 representative isolates from Guangdong and Hainan were different from those in J56P, J199C, and WF. The N‐ and C‐terminals of CP are related to the long‐distance movement, viral particle assembly, and interaction with the 3′‐UTR of the virus (Dolja et al., 1995; Jagadish et al., 1991; Rojas et al., 1997; Varrelmann & Maiss, 2000; Yusibov et al., 1996; Zamora et al., 2017). A point mutation from Ser47 to Pro of pea seed‐borne mosaic virus or from Asp5 to Lys of tobacco vein mottling virus in the N‐terminal of CP alters the systematic spread ability of viruses in *Chenopodium quinoa* or tobacco, respectively (Andersen & Johansen, 1998; López‐Moya & Pirone, 1998). A single substitution (Ser7 to Gly) at the CP N‐terminal of potato virus A reduces virus accumulation by 10‐fold but does not affect the rate of systematic transportation (Andrejeva et al., 1999). In the present study, most of the variation sites were located at the N‐ or C‐terminals of CP. Given that China and Japan isolates are much different in terms of virus hosts, we speculate that those variations may contribute to difference of the host range. This study also found that one site (Asn117 to Asp) of Guangdong and Hainan isolates was inconsistent, which provides a basis for distinguishing isolates from the two regions.

Among the indicators for studying species diversity, the haplotype diversity index (*Hd*) and nucleotide diversity index (*Pi*) are widely used. *Hd* refers to the frequency of two different haplotypes randomly selected in sample (Excoffier et al., 1992), and *Pi* refers to the average number of nucleotide differences or substitutions of a set of DNA sequence unit sites in a population (Nei & Li, 1979). Grant and Bowen (1998) divide a virus population into four types according to different combinations of *Hd* and *Pi*. The first type is the lower *Hd* (<0.5) and *Pi* (<0.005), indicating that the population has recently had a bottleneck effect or a founder effect by a single, minority lineage. The second type is high *Hd* and low *Pi*, indicating that the population experiences sudden rapid propagation of remaining good individuals after a sharp decrease. The third type is low *Hd* and high *Pi*, indicating that a large population of individuals has sharply reduced to a small population or two populations with distant relationships. The fourth type is high *Hd* and high *Pi*, indicating that the two populations have developed from the same population after a long period of expansion. In the present study, the populations of PLDMV from Guangdong and Hainan belong to the second type (i.e., high *Hd* and low *Pi*), which indicates that the populations from two regions have experienced rapid population growth and accumulated new mutations after the bottleneck effect. This result may explain why PLDMV rapidly expanded in Guangdong and Hainan for recent years, resulting in widespread damage to transgenic papaya.

A phylogenetic analysis of PLDMV CP nucleotide sequences showed that all of the PLDMV isolates were distinctly divided into two groups (Figure 5). Although Guangdong and Hainan isolates were divided into different subgroups on the basis of geographical locations, they all belong to group II, whereas J56P, J199C, WF (Bau et al., 2008; Maoka et al., 1995), and other Japan isolates and Taiwan China isolates were classified as group I. The genetic distance values of the isolates between group II and group I were 0.046 ± 0.006, which was significantly greater than that within in groups I or II, which was 0.040 ± 0.005 or 0.005 ± 0.001, respectively. This result suggests that PLDMV isolates from Guangdong and Hainan have evolved into a new lineage.

Recombination is an important factor to promote virus evolution, which can increase genomic biodiversity (Dietrich et al., 2007), reduce mutations in specific genome sequences (Worobey & Holmes, 1999), and restore genome integrity (Garcíaarenal et al., 2001). In addition, recombination enhances the virulence of the virus and extends its host ranges (Escriu et al., 2007). In the present study, obvious recombination was not found in 111 PLDMV isolates from Guangdong and Hainan. However, these isolate samples were collected from 2012 to 2019. In fact, as early as 2012, the disease had occurred in some papaya plantations in Hainan Province (unpublished data). Whether early isolates have evolved through recombination mutations and whether rapid population growth has occurred after the bottleneck effect remain to be confirmed by further research in the future.

In the present study, the PLDMV CP sequences of Guangdong and Hainan isolates exhibited a high degree of genetic diversity. Three indicator values were used to test the population differentiation between the Guangdong and Hainan isolates on the basis of CP nucleotide sequences. *K*_ST_ and *F*_ST_ may measure the relative proportions of total genetic diversity attributable to among populations and range from 0.00 to 1.00. A value of 1.00 for *K_ST_* or *F_ST_* indicates that populations are completely differentiated, whereas a value of 0.00 indicates that the populations are identical (Hudson et al., 1992). *K*_ST_ or *F*_ST_ values between 0.15 or 0.25 indicate high population differentiation, and values >0.25 indicate very high genetic differentiation among populations (Balloux & Moulin, 2002). *S*_nn_ may indicate the frequency of the most similar sequences in the same population; *S_nn_* values close to 1.0 indicate that the population is highly differentiated, whereas values near 0.5 (Hudson, 2000) indicate that the population is identical. In the current study, the *K*_ST_ and *F*_ST_ values were close to or higher than 0.25, and the *S*_nn_ values were close to 1.00, suggesting that the Guangdong and Hainan isolates are composed of a highly differentiated new population of PLDMV in South China. When DNA fragments have little or no recombination, the *N*_m_ value is used to determine whether gene flow occurs between populations (Whitlock & Mccauley, 1999). When *N*_m_ < 1, genetic drift between groups is prone to occur, gene flow between groups is not frequent, and genetic differentiation is large; when *N*_m_ > 1, gene flow can occur between groups through some channel or the geographical distance between two groups is within the group. In the present study, the *N*_m_ value of PLDMV populations in Guangdong and Hainan was less than 1, indicating that the gene flow between populations is infrequent and that genetic differentiation is obvious, which is consistent with the high genetic differentiation between PLDMV populations obtained from *K*_ST_ or *F*_ST_ values. Given that Guangdong and Hainan provinces are separated by the Qiongzhou Strait, they may have relatively limited migration and diffusion opportunities between different geographical populations, thus limiting gene exchange between populations to some extent.

The positive selection of viral population may endow the virus increased fitness to adapt to new hosts or environments, whereas rapid divergence driven by positive selection has been seldom demonstrated (Nielsen, 1999). Similar to other viral gene sequences, a majority of the codons in the PLDMV CP sequences of the Guangdong and Hainan isolates in the present study were detected to be under the status of negative (purifying) selection. This result suggests that most of the codon mutations in the PLDMV genome are detrimental and are thus easily eliminated by natural selection. In this case, the choice may come from differences in living environment between the two geographical regions, such as differences in papaya cultivars and climate conditions. After a long‐term follow‐up survey of genetically modified cultivars in Guangdong and Hainan provinces, we found that “Huanong No. 1” is the dominant cultivar grown in Guangdong Province, whereas various cultivars from Taiwan China and other regions, including “Huanong No. 1,” are grown in Hainan Province. Guangdong Province is located at 20°13′N–25°31′N, 109°39′E–117°19′E, and Hainan Province is located at 19°20′N–20°10′N, 108°21′E–111°03′E. The former has a subtropical climate and a tropical monsoon climate with an average annual temperature of 19–23°C, while the latter is a tropical monsoon climate with an annual average temperature of 23–25°C. The two regions were separated by the Qiongzhou Strait, resulting in certain geographical distance (Du et al., 2013). These differences may lead to the gradual differentiation of Guangdong and Hainan isolates and eventually induce those isolates to evolve into two subgroups.

Tajima (1989) has developed a statistical method for testing the neutral mutation hypothesis by using the average number of nucleotide differences and the number of segregating sites. If a population experiences a bottleneck or balance, Tajima's *D* value is significantly higher than zero. If a population experiences a size expansion or directional selection, Tajima's *D* value is significantly less than zero. Given that both balance and directional selection fall into the category of positive selection, natural selection may be accepted as long as the Tajima's *D* value deviates significantly from zero; however, neutral selection cannot be rejected when the Tajima's *D* value does not significantly deviate from zero. Fu and Li (1993) proposed Fu and Li's *D* and Fu and Li's *F* test for neutral selection in comparison with Tajima's *D* test. The proposed method considers the availability of external branches so that a rooted tree can be constructed for a given set of DNA sequences. The number of external mutations is different from its neutral expected values in the presence of selection, whereas the number of internal mutations is only slightly affected by the presence of selection. In the present study, Tajima's *D*, Fu and Li's *D*, and Fu and Li's *F* values from Guangdong and Hainan isolates were all negative, and the data showed significant (0.01 ≤ *p *< .05) or extremely significant (0.01 ≤ *p *< .05) differences between Guangdong and Hainan populations, respectively. These results revealed that the populations of the two regions deviated from the neutral evolution, which implied they had experienced population expansion events in the past and may still be unstable populations.

In summary, we analyzed and confirmed the population characteristics of PLDMV isolates in South China by collecting transgenic “Huanong No.1” papaya samples from Guangdong and Hainan in 2012–2019. These isolates are highly differentiated from the previously reported isolates of Japan and Taiwan China and therefore have led to the formation of a new PLDMV group that can infect genetically engineered papaya grown widely in South China. Notably, the emergence of PLDMV lineage in commercialized PRSV‐resistant transgenic papaya indicates a huge threat to the papaya industry in South China. Antiviral research must be urgently carried out to solve the problem of the disease.

## CONFLICT OF INTERESTS

The authors declare that there are no competing interests.

## AUTHOR CONTRIBUTION

**Cuiping Mo:** Data curation (lead); Formal analysis (lead); Investigation (lead); Software (lead); Writing‐original draft (equal); Writing‐review & editing (equal). **Zilin Wu:** Data curation (equal); Investigation (lead); Methodology (lead); Resources (equal); Software (equal). **Hengping Xie:** Data curation (equal); Investigation (equal); Resources (equal). **Shuguang Zhang:** Investigation (equal); Methodology (equal); Resources (equal). **Huaping Li:** Conceptualization (lead); Funding acquisition (lead); Supervision (lead); Writing‐original draft (lead); Writing‐review & editing (lead).

## Supporting information

Table S1

## Data Availability

These gene sequences of isolates in the present study have been submitted to GenBank, and accession numbers (MN840853–MN840963) are accessible directly at the individual records in National Center for Biotechnology Information (https://www.ncbi.nlm.nih.gov).
