# Supplementary material for: Genetic diversity analysis of papaya leaf distortion mosaic virus isolates infecting transgenic papaya “Huanong No. 1” in South China
Source: Ecol Evol. 2020 Sep 22;10(20):11671–83. doi: 10.1002/ece3.6800 (PMC7593138; doi:10.1002/ece3.6800)
Supplement: Supplementary file 1 — Table S1 [file ECE3-10-11671-s001.doc]

Appendix Table S1: PLDMV isolates in this study

| PLDMV isolates in this study | | | | | |
| --- | --- | --- | --- | --- | --- |
| Isolates | Country | Host | Date | Accession Number | Reference |
| GZ19 | China: Guangdong, Guangzhou | *Carica papaya* | 2016 | MN840883 | This study |
| GZ20 | China: Guangdong, Guangzhou | *Carica papaya* | 2016 | MN840882 | This study |
| GZ23 | China: Guangdong, Guangzhou | *Carica papaya* | 2016 | MN840877 | This study |
| GZ24 | China: Guangdong, Guangzhou | *Carica papaya* | 2016 | MN840876 | This study |
| GZ25 | China: Guangdong, Guangzhou | *Carica papaya* | 2016 | MN840866 | This study |
| GZ26 | China: Guangdong, Guangzhou | *Carica papaya* | 2016 | MN840865 | This study |
| GZ27 | China: Guangdong, Guangzhou | *Carica papaya* | 2016 | MN840875 | This study |
| GZ28 | China: Guangdong, Guangzhou | *Carica papaya* | 2016 | MN840881 | This study |
| GZ29 | China: Guangdong, Guangzhou | *Carica papaya* | 2016 | MN840880 | This study |
| GZ30 | China: Guangdong, Guangzhou | *Carica papaya* | 2016 | MN840885 | This study |
| GZ31 | China: Guangdong, Guangzhou | *Carica papaya* | 2016 | MN840879 | This study |
| GZ32 | China: Guangdong, Guangzhou | *Carica papaya* | 2016 | MN840874 | This study |
| GZ33 | China: Guangdong, Guangzhou | *Carica papaya* | 2016 | MN840884 | This study |
| GZ34 | China: Guangdong, Guangzhou | *Carica papaya* | 2016 | MN840878 | This study |
| GZ37 | China: Guangdong, Guangzhou | *Carica papaya* | 2016 | MN840873 | This study |
| GZ38 | China: Guangdong, Guangzhou | *Carica papaya* | 2016 | MN840872 | This study |
| GZ39 | China: Guangdong, Guangzhou | *Carica papaya* | 2016 | MN840871 | This study |
| GZ40 | China: Guangdong, Guangzhou | *Carica papaya* | 2016 | MN840870 | This study |
| GZ41 | China: Guangdong, Guangzhou | *Carica papaya* | 2016 | MN840869 | This study |
| GZ42 | China: Guangdong, Guangzhou | *Carica papaya* | 2016 | MN840868 | This study |
| NSA3 | China: Guangdong, Guangzhou | *Carica papaya* | 2016 | MN840867 | This study |
| NS60 | China: Guangdong, Guangzhou | *Carica papaya* | 2017 | MN840862 | This study |
| NS62 | China: Guangdong, Guangzhou | *Carica papaya* | 2017 | MN840863 | This study |
| NS79 | China: Guangdong, Guangzhou | *Carica papaya* | 2017 | MN840864 | This study |
| FM80 | China: Guangdong, Guangzhou | *Carica papaya* | 2017 | MN840853 | This study |
| FM81 | China: Guangdong, Guangzhou | *Carica papaya* | 2017 | MN840854 | This study |
| FM82 | China: Guangdong, Guangzhou | *Carica papaya* | 2017 | MN840855 | This study |
| FM83 | China: Guangdong, Guangzhou | *Carica papaya* | 2017 | MN840860 | This study |
| FM84 | China: Guangdong, Guangzhou | *Carica papaya* | 2017 | MN840861 | This study |
| FM185 | China: Guangdong, Guangzhou | *Carica papaya* | 2018 | MN840858 | This study |
| FM186 | China: Guangdong, Guangzhou | *Carica papaya* | 2018 | MN840859 | This study |
| NS194 | China: Guangdong, Guangzhou | *Carica papaya* | 2019 | MN840856 | This study |
| NS195 | China: Guangdong, Guangzhou | *Carica papaya* | 2019 | MN840857 | This study |
| SD11 | China: Hainan, Sanya | *Carica papaya* | 2012 | MN840924 | This study |
| SD12 | China: Hainan, Sanya | *Carica papaya* | 2012 | MN840904 | This study |
| SD40 | China: Hainan, Dongfang | *Carica papaya* | 2012 | MN840903 | This study |
| SD41 | China: Hainan, Dongfang | *Carica papaya* | 2012 | MN840902 | This study |
| SD42 | China: Hainan, Dongfang | *Carica papaya* | 2012 | MN840963 | This study |
| SD43 | China: Hainan, Dongfang | *Carica papaya* | 2012 | MN840923 | This study |
| SD44 | China: Hainan, Dongfang | *Carica papaya* | 2012 | MN840951 | This study |
| SD45 | China: Hainan, Dongfang | *Carica papaya* | 2012 | MN840901 | This study |
| SD46 | China: Hainan, Dongfang | *Carica papaya* | 2012 | MN840900 | This study |
| SD47 | China: Hainan, Dongfang | *Carica papaya* | 2012 | MN840899 | This study |
| SD48 | China: Hainan, Dongfang | *Carica papaya* | 2012 | MN840922 | This study |
| SD49 | China: Hainan, Dongfang | *Carica papaya* | 2012 | MN840898 | This study |
| SD54 | China: Hainan, Dongfang | *Carica papaya* | 2012 | MN840897 | This study |
| SD55 | China: Hainan, Dongfang | *Carica papaya* | 2012 | MN840896 | This study |
| SD56 | China: Hainan, Ledong | *Carica papaya* | 2012 | MN840921 | This study |
| S3 | China: Hainan, Sanya | *Carica papaya* | 2014 | MN840905 | This study |
| S4 | China: Hainan, Sanya | *Carica papaya* | 2014 | MN840943 | This study |
| S5 | China: Hainan, Sanya | *Carica papaya* | 2014 | MN840942 | This study |
| S6 | China: Hainan, Sanya | *Carica papaya* | 2014 | MN840941 | This study |
| S7 | China: Hainan, Sanya | *Carica papaya* | 2014 | MN840940 | This study |
| S8 | China: Hainan, Sanya | *Carica papaya* | 2014 | MN840939 | This study |
| S9 | China: Hainan, Sanya | *Carica papaya* | 2014 | MN840938 | This study |
| S10 | China: Hainan, Sanya | *Carica papaya* | 2014 | MN840937 | This study |
| S12 | China: Hainan, Sanya | *Carica papaya* | 2014 | MN840956 | This study |
| S13 | China: Hainan, Sanya | *Carica papaya* | 2014 | MN840936 | This study |
| S18 | China: Hainan, Ledong | *Carica papaya* | 2014 | MN840955 | This study |
| S19 | China: Hainan, Ledong | *Carica papaya* | 2014 | MN840887 | This study |
| S20 | China: Hainan, Changjiang | *Carica papaya* | 2014 | MN840886 | This study |
| S21 | China: Hainan, Sanya | *Carica papaya* | 2014 | MN840935 | This study |
| S22 | China: Hainan, Sanya | *Carica papaya* | 2014 | MN840954 | This study |
| S24 | China: Hainan, Sanya | *Carica papaya* | 2014 | MN840953 | This study |
| S27 | China: Hainan, Ledong | *Carica papaya* | 2014 | MN840960 | This study |
| S28 | China: Hainan, Ledong | *Carica papaya* | 2014 | MN840952 | This study |
| S30 | China: Hainan, Ledong | *Carica papaya* | 2014 | MN840888 | This study |
| HA2 | China: Hainan, Sanya | *Carica papaya* | 2016 | MN840961 | This study |
| HA4 | China: Hainan, Sanya | *Carica papaya* | 2016 | MN840950 | This study |
| HA5 | China: Hainan, Sanya | *Carica papaya* | 2016 | MN840959 | This study |
| HA6 | China: Hainan, Sanya | *Carica papaya* | 2016 | MN840949 | This study |
| HA7 | China: Hainan, Sanya | *Carica papaya* | 2016 | MN840958 | This study |
| HA8 | China: Hainan, Sanya | *Carica papaya* | 2016 | MN840948 | This study |
| HA9 | China: Hainan, Sanya | *Carica papaya* | 2016 | MN840947 | This study |
| HA10 | China: Hainan, Sanya | *Carica papaya* | 2016 | MN840946 | This study |
| HA20 | China: Hainan, Ledong | *Carica papaya* | 2016 | MN840934 | This study |
| HA21 | China: Hainan, Ledong | *Carica papaya* | 2016 | MN840920 | This study |
| HA22 | China: Hainan, Ledong | *Carica papaya* | 2016 | MN840933 | This study |
| HA23 | China: Hainan, Ledong | *Carica papaya* | 2016 | MN840945 | This study |
| HA24 | China: Hainan, Ledong | *Carica papaya* | 2016 | MN840932 | This study |
| HA25 | China: Hainan, Ledong | *Carica papaya* | 2016 | MN840931 | This study |
| HA26 | China: Hainan, Ledong | *Carica papaya* | 2016 | MN840944 | This study |
| HA27 | China: Hainan, Ledong | *Carica papaya* | 2016 | MN840919 | This study |
| HA28 | China: Hainan, Ledong | *Carica papaya* | 2016 | MN840918 | This study |
| HA31 | China: Hainan, Dongfang | *Carica papaya* | 2016 | MN840917 | This study |
| HA34 | China: Hainan, Dongfang | *Carica papaya* | 2016 | MN840962 | This study |
| HA35 | China: Hainan, Dongfang | *Carica papaya* | 2016 | MN840930 | This study |
| HA38 | China: Hainan, Dongfang | *Carica papaya* | 2016 | MN840929 | This study |
| HA39 | China: Hainan, Dongfang | *Carica papaya* | 2016 | MN840928 | This study |
| HA40 | China: Hainan, Dongfang | *Carica papaya* | 2016 | MN840895 | This study |
| HA41 | China: Hainan, Dongfang | *Carica papaya* | 2016 | MN840916 | This study |
| HA42 | China: Hainan, Dongfang | *Carica papaya* | 2016 | MN840894 | This study |
| HA45 | China: Hainan, Dongfang | *Carica papaya* | 2016 | MN840893 | This study |
| HA47 | China: Hainan, Dongfang | *Carica papaya* | 2016 | MN840915 | This study |
| HA50 | China: Hainan, Dongfang | *Carica papaya* | 2016 | MN840927 | This study |
| HA51 | China: Hainan, Dongfang | *Carica papaya* | 2016 | MN840926 | This study |
| HA52 | China: Hainan, Ledong | *Carica papaya* | 2016 | MN840957 | This study |
| HA53 | China: Hainan, Ledong | *Carica papaya* | 2016 | MN840892 | This study |
| HA54 | China: Hainan, Ledong | *Carica papaya* | 2016 | MN840914 | This study |
| HA55 | China: Hainan, Ledong | *Carica papaya* | 2016 | MN840913 | This study |
| HA56 | China: Hainan, Ledong | *Carica papaya* | 2016 | MN840891 | This study |
| HA57 | China: Hainan, Ledong | *Carica papaya* | 2016 | MN840890 | This study |
| HA58 | China: Hainan, Ledong | *Carica papaya* | 2016 | MN840889 | This study |
| HA59 | China: Hainan, Ledong | *Carica papaya* | 2016 | MN840912 | This study |
| HA60 | China: Hainan, Ledong | *Carica papaya* | 2016 | MN840911 | This study |
| HA61 | China: Hainan, Ledong | *Carica papaya* | 2016 | MN840910 | This study |
| HA62 | China: Hainan, Ledong | *Carica papaya* | 2016 | MN840909 | This study |
| HA63 | China: Hainan, Ledong | *Carica papaya* | 2016 | MN840908 | This study |
| HA64 | China: Hainan, Ledong | *Carica papaya* | 2016 | MN840907 | This study |
| HA65 | China: Hainan, Sanya | *Carica papaya* | 2016 | MN840906 | This study |
| HA66 | China: Hainan, Sanya | *Carica papaya* | 2016 | MN840925 | This study |
| DF_HN | China: Hainan, Dongfang | *Carica papaya* | 2012 | JX974555.1 | (Tuo et al., 2013) |
| LM_HN | China: Hainan, Lingshui | *Carica papaya* | 2015 | KT633944.1 | Unpublished |
| KS_TW | China: Taiwan | *Carica papaya* | 2007 | EU233272.1 | Unpublished |
| WF_TW | China: Taiwan | *Carica papaya* | 2007 | EF675245.1 | (Bau et al., 2008) |
| TD_TW | China: Taiwan | *Carica papaya* | 2007 | EU240890.1 | (Bau et al., 2008) |
| ZS_TW | China: Taiwan | *Carica papaya* | 2007 | EU240889.1 | (Bau et al., 2008) |
| CZ_TW | China: Taiwan, Pingtong | *Carica papaya* | 2012 | JX416282.1 | Unpublished |
| J56P_JP | Japan: Okinawa, Ishigaki | *Carica papaya* | 1990 | AB088221.1 | (Maoka et al., 1996) |
| J69P_JP | Japan: Okinawa | *Carica papaya* | 1991 | AB092814.1 | (Maoka and Hataya, 2005) |
| J179P_JP | Japan: Okinawa, Ishigaki | *Carica papaya* | 1992 | AB092815.1 | (Maoka and Hataya, 2005) |
| J199C_JP | Japan: Okinawa, Ishigaki | *Cucumis sativus* | 1995 | AB092816.1 | (Maoka and Hataya, 2005) |
